# Pulse Compression Shape-Based ADC/DAC Chain Synchronization Measurement Algorithm with Sub-Sampling Resolution

**DOI:** 10.3390/s24092831

**Published:** 2024-04-29

**Authors:** Xiangyu Hao, Hongji Fang, Wei Luo, Bo Zhang

**Affiliations:** 1 College of Biomedical Engineering and Instrument Science, Zhejiang University, Hangzhou 310027, China; 12215046@zju.edu.cn (X.H.); 11815025@zju.edu.cn (H.F.); luo.wei@zju.edu.cn (W.L.); 2 College of Information Science and Electronic Engineering, Zhejiang University, Hangzhou 310027, China

**Keywords:** delay parameter measurement, multi-channel system synchronization, pulse compression, sub-sampling resolution

## Abstract

In this article, we address the problem of synchronizing multiple analog-to-digital converter (ADC) and digital-to-analog converter (DAC) chains in a multi-channel system, which is constrained by the sampling frequency and inconsistencies among the components during system integration. To evaluate and compensate for the synchronization differences, we propose a pulse compression shape-based algorithm to measure the entire delay parameter of the ADC/DAC chain, which achieves sub-sampling resolution by mapping the shape of the discrete pulse compression peak to the signal propagation delay. Moreover, owing to the matched filtering in the pulse compression process, the algorithm exhibits good noise performance and is suitable for wireless scenarios. Experiments verified that the algorithm can achieve precise measurements with sub-sampling resolution in scenarios where the signal-to-noise ratio (SNR) is greater than −10 dB.

## 1. Introduction

Multi-channel systems integrate multiple ADCs and DACs, allowing for independent signal acquisition or generation tasks. For instance, Multiple-Input Multiple-Output (MIMO) systems employ multiple ADC/DAC chains for data transmission to improve efficiency [1]. They also utilize spatial and temporal coding and decoding techniques to enhance system reliability and interference resistance [2,3,4,5]. Additionally, such multi-channel systems can alternate the sampling of the same signal to achieve a higher sampling rate [6] and the time-interleaved sampling model is the mainstream implementation architecture for high-sampling-rate converters and systems in the current context [7,8,9]. Furthermore, multi-channel systems are widely applied in monitoring scenarios for biomedical signals such as electrocardiography (ECG) [10] and electroencephalography (EEG) [11].

The synchronization issues between channels in the aforementioned multi-channel systems have been the subjects of extensive attention from both the academic and industrial communities [12,13,14]. Moreover, the synchronization performance of signal sampling and data transmission across different channels has a significant impact on the reliability of the system. For instance, in ultra-wideband pulse radio location and navigation systems, the principle relies on the time differences of arrival of synchronized signals across multiple channels [15]. Asynchrony between channels will accumulate and result in significant deviations in the positioning distance [16,17]. Additionally, in signal acquisition scenarios, asynchrony between channels can introduce mismatch errors that affect system performance or reduce the accuracy of signal analysis [18,19,20].

In the past, researchers have proposed a series of solutions to address the synchronization issues in multi-channel systems. The synchronization schemes proposed in references [21,22] are based on Bluetooth Low Energy (BLE) technology and utilize timestamps to achieve simultaneous ADC sampling and data transmission alignment. This approach relies on an external synchronization source, and the limited performance of the microcontroller restricts the synchronization accuracy. To meet the synchronization requirements of current high-performance systems, research efforts have been focused on internal design aspects [23,24,25]. Scholars have proposed a daisy chain structure that utilizes the internal frequency division system of high-speed converters to achieve synchronization through the propagation of the synchronization signal [26]. Engineers commonly utilize a homologous clock source and rigorously control clock routing delays to enhance synchronization performance [27]. Meanwhile, some researchers have calibrated the mismatch error of the time-interleaved converters [28,29,30] and the acquisition system [31] based on multi-channel sampling synchronization.

As the synchronization performance of multi-channel sampling improves, significant breakthroughs have been made in data transmission technologies. The JESD204 protocol, proposed by JEDEC, has gradually replaced CMOS and LVDS as the mainstream data transmission solution between converter devices and logic devices [32]. Researchers have achieved multi-channel data transmission alignment by utilizing the system reference edge [33] and the deterministic latency characteristics of the JESD204B/C protocols [34,35,36]. In addition, some researchers have alleviated the pressure of synchronous design by integrating converters and radio frequency components [37,38,39].

However, multi-channel synchronization issues still exhibit uncertainty at the system level. A complete ADC/DAC chain comprises multiple elements, such as converters, conditioning circuits, and connecting cables. For high-precision synchronization requirements, inconsistencies among the components of different channels can lead to significant deviations [40]. Therefore, researchers usually need to design an additional calibration scheme during system integration to address these inconsistencies. Additionally, engineers need to measure and compensate for the parameters of each component using vector network analyzers. However, these methods are expensive and difficult to implement on a large scale. To address this issue, we present a pulse compression shape-based ADC/DAC chain synchronization measurement algorithm and our contributions are summarized as follows:

- We propose an algorithm that can directly obtain the delay parameters of the entire ADC/DAC chain without the need for any additional hardware expenses.
- We map the location of the pulse compression peak within the calculation window to the delay parameters and model the discretized peak shape to achieve sub-sampling resolution. This breakthrough enables us to overcome the limitation of sampling frequency on the accuracy of digital signal processing.
- The algorithm exhibits good noise performance. In typical wireless scenarios with an SNR greater than 10 dB, the results can be accurately measured up to 0.01 sampling points. Additionally, its resolution can still be maintained at 0.1 sampling points in high-noise scenarios with an SNR ranging between 10 dB and −10 dB.
- We conducted a validation of the algorithm’s effectiveness and precision by designing a multi-channel radio platform. The results indicate that it can accurately measure the delay values of the ADC/DAC chains, making it widely applicable for optimizing the synchronization performance in multi-channel systems.

The remainder of this article is organized as follows. Section 2 introduces the theory of pulse compression and provides a detailed description of the principles behind the ADC/DAC chain synchronization measurement algorithm. In Section 3, an analysis of the algorithm’s computational errors and noise performance is presented. Additionally, Section 4 further validates the algorithm on a multi-channel radio platform. Finally, Section 5 provides a summary of the findings and conclusions presented throughout the article.

## 2. Methods

### 2.1. Pulse Compression Theory

Modulation methods commonly used in pulse compression radar transmitters include polyphase codes, Costas codes, Barker codes, and frequency-stepping [41]. The most widely used method is linear frequency modulation (LFM), which was invented by R.H. Dickie in 1945 [42]. The waveform of an LFM signal with bandwidth *B* and duration *T* can be expressed as

(1) $$S_{lfm}\left(t\right)=A\cdot \mathrm{rect}\left(\frac{t}{T}\right)\cdot e^{2\pi j\left(f_{c}t+\frac{1}{2}\mu t^{2}\right)},$$

where *A* represents the amplitude of the LFM signal, rect() represents the rectangular window function, $f_{c}$ is the carrier frequency, and μ is the rate of change of the frequency, which can be calculated via

(2) $$\mu =\frac{B}{T}.$$

Pulse compression essentially involves the application of matched filtering to a modulated signal. The impulse response of the matched filter is a conjugated time-reversed version of the transmitted signal [43], and it is expressed as

(3) $$H\left(t\right)=K\cdot S_{lfm}^{*}\left(t_{0}-t\right),$$

where *K* represents the attenuation coefficient of the filter and $t_{0}$ represents the physical delay to the signal caused by the matched filter. The matched filtering process is mathematically equivalent to calculating the correlation between the received and transmitted signals. The result of the matched filtering is represented by $S_{pulse}\left(t\right)$, which is expressed as

(4) $$S_{pulse}\left(t\right)=\int_{-\infty }^{\infty }S_{lfm}\left(x\right)\cdot H\left(t-x\right)dx.$$

We mathematically assume

(5) $$t-t_{0}=t^{\prime }.$$

Substituting Equations (1) and (3) into Equation (4) yields

(6) $$\begin{array}{cc} S_{pulse}\left(t^{\prime }\right)= & K\cdot \int_{-\infty }^{\infty }S_{lfm}\left(x\right)\cdot S_{lfm}^{*}\left(x-t^{\prime }\right)dx\\ = & KA^{2}T\cdot \mathrm{rect}\left(\frac{t^{\prime }}{2T}\right)\cdot \frac{\sin\left(\pi \mu T\left(1-\frac{\left|t^{\prime }\right|}{T}\right)t^{\prime }\right)}{\pi \mu Tt^{\prime }}\cdot e^{2\pi jf_{c}t^{\prime }} \end{array}.$$

In practice, we preselect the parameters of the LFM signal, such as *A*, μ, *T*, and $f_{c}$. The attenuation coefficient *K* of the matched filter can also be easily obtained. Equation (6) provides a detailed expression for the pulse compression result. When the condition $t^{\prime }<<T$ is satisfied, its envelope curve can be approximated as

(7) $$S_{pulse}\left(t^{\prime }\right)=KA^{2}T\cdot \mathrm{rect}\left(\frac{t^{\prime }}{2T}\right)\cdot \mathrm{Sa}\left(\pi \mu Tt^{\prime }\right).$$

Equation (7) features a significant peak, the location of which depends on $t^{\prime }$. This provides us with a theoretical basis for implementing the measurement of chain delay parameters.

### 2.2. Algorithm Description

The basic principles of the algorithm proposed in this article are as follows. The radio system sends a specific LFM signal through a DAC. Then, the electromagnetic wave propagates through a specific path and is sampled by the ADC for matched filtering operation. The filter matches the signal on the same timescale and accumulates the energy of the long-duration LFM signal at the peak. The location of the pulse compression peak in the calculation window is linearly related to the propagation delay of the signal in the ADC/DAC chains.

**Figure 1:**
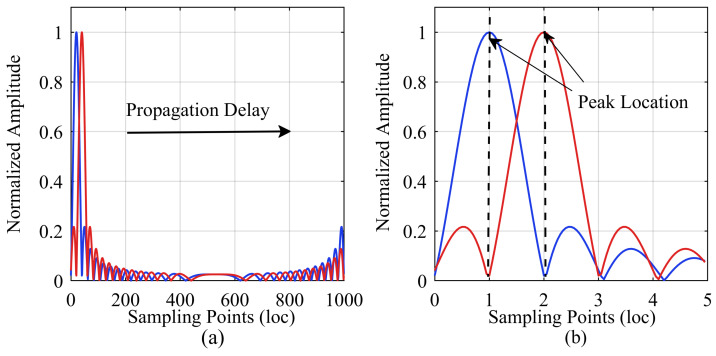
Peak shifting in the pulse compression calculation window. (**a**) Overall schematic; (**b**) Local zoom-in illustration.

Figure 1 shows an example of pulse compression peak shifting. The blue line represents a direct connection between the ADC and DAC interfaces without any electromagnetic wave propagation delay, while the red line represents the introduction of a propagation delay into the chain. When the chain is extended, the propagation delay increases. The pulse compression peak moves in the direction indicated by the arrow in Figure 1a. Because the LFM signal is discretized after sampling, the smallest unit of peak movement is a sampling point, as shown in Figure 1b. This implies that the resolution of the measurement is limited by the system’s sampling frequency. For the convenience of expression, we denote a sampling point unit as loc.

**Figure 2:**
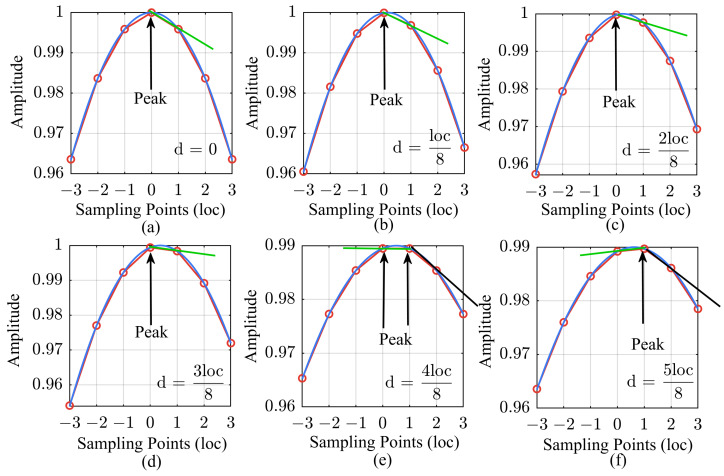

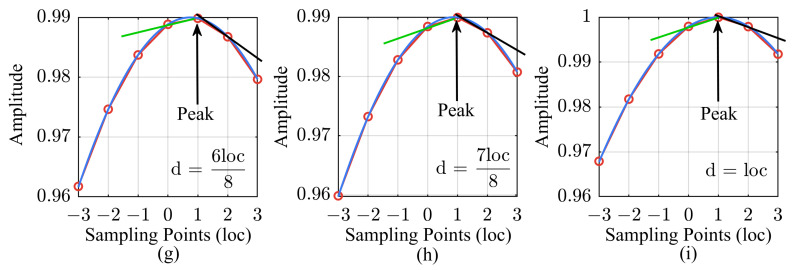
Schematic diagrams of the sampling resolution measurement errors and sub-sampling resolution mapping. (**a**) Propagation delay: 0; (**b**) Propagation delay: $\frac{\mathrm{loc}}{8}$; (**c**) Propagation delay: $\frac{2\mathrm{loc}}{8}$; (**d**) Propagation delay: $\frac{3\mathrm{loc}}{8}$; (**e**) Propagation delay: $\frac{4\mathrm{loc}}{8}$; (**f**) Propagation delay: $\frac{5\mathrm{loc}}{8}$; (**g**) Propagation delay: $\frac{6\mathrm{loc}}{8}$; (**h**) Propagation delay: $\frac{7\mathrm{loc}}{8}$; (**i**) Propagation delay: loc. The signal amplitudes in the figure have been normalized.

Owing to the limitations of the sampling frequency, it is usually challenging for the resolution at the sampling level to meet the synchronization measurement requirements of ADC/DAC chains in a high-performance radio system. The delay of the electromagnetic wave propagation in the system varies continuously, which introduces errors at the sub-sampling level into the results. To demonstrate this phenomenon intuitively, we select $\frac{\mathrm{loc}}{8}$ as the smallest unit of variation in the propagation delay, as shown in Figure 2. The blue curves in the figure show the actual movement of the pulse compression peak, whereas the red dots represent the results obtained from digital system computations. This difference is precisely due to sampling discretization. As shown in Figure 2e, when the peak value is exactly between two sampling points, the maximum measurement error is $\frac{\mathrm{loc}}{2}$, and the determination of the peak sampling point is ambiguous.

In this article, we map the shape of the discretized pulse compression peak to the sub-sampling level measurements. The method is based on the slope between the highest point of the peak and the next sampling point, as indicated by the straight line in Figure 2. To perform the mapping calculations, the amplitude of the pulse compression results is normalized to obtain

(8) $$S_{peak}\left(t\right)=\frac{\sin(\pi \mu Tt)}{\pi \mu Tt},$$

We define the variable *x* as

(9) $$x=t\cdot F_{s},$$

where $F_{s}$ represents the sampling frequency of the system. The physical meaning of *x* is similar to that of the sampling point, with the only difference being that *x* is a continuous variable. Substituting Equations (2) and (9) into Equation (8) yields

(10) $$S_{peak}\left(x\right)=\frac{\sin\left(\pi \frac{B}{F_{s}}x\right)}{\pi \frac{B}{F_{s}}x}.$$

The fitting function is defined as

(11) $$S_{fit}\left(x\right)=\frac{\sin\alpha x}{\alpha x}-\frac{\sin\alpha (x+1)}{\alpha (x+1)},$$

where α is a parameter that can be calculated via

(12) $$\alpha =\pi \frac{B}{F_{s}}.$$

As the position of the main peak shifts to the next sampling point with a step smaller than a sampling point, the value of *x* changes from 0 to −1. The result calculated using Equation (11) is the slope between the two sampling points, which is represented by the green line in Figure 2. This completes the mapping of the peak’s change in shape to the sub-sampling resolution measurement. When the determination of the peak sampling point changes, the mapping relationship must be adjusted accordingly. For example, the mapping slope in the case of $d>\frac{\mathrm{loc}}{2}$ is represented by the black line instead of the green line. Therefore, the value of *x* now ranges from 0 to −0.5 in the case of $d\leq \frac{\mathrm{loc}}{2}$, and it changes from 0.5 to 0 in the case of $d>\frac{\mathrm{loc}}{2}$.

Considering the complexity of solving the function $S_{fit}\left(x\right)$, applying the third-order Taylor expansion to Equation (11) gives

(13) $$S_{fit}\left(x\right)=\frac{20\alpha^{2}-\alpha^{4}}{120}+\left(\frac{\alpha^{2}}{3}-\frac{\alpha^{4}}{30}\right)x-\frac{\alpha^{4}}{20}x^{2}-\frac{\alpha^{4}}{30}x^{3}+o\left(x^{k}\right),$$

where k=4,5,6⋯. In practical engineering applications, the sampling frequency is generally selected to be 5–10 times the bandwidth of the signal. This implies that the quadratic and cubic terms in Equation (13) can be approximated as infinitesimal quantities, and the function can be further simplified as

(14) $$S_{fit}\left(x\right)=\frac{20\alpha^{2}-\alpha^{4}}{120}+\left(\frac{\alpha^{2}}{3}-\frac{\alpha^{4}}{30}\right)x+o\left(x^{k}\right),$$

where k=2,3,4,5,6⋯. The entire process of the synchronization delay measurement algorithm is illustrated in Algorithm 1.
**Algorithm 1** Synchronization Delay Measurement.**Input:** Signal $S_{lfm}\left[n\right]$, Filter H[n], Duration *N*.**Output:** Synchronization delay value *D*.1: $N_{fft}\leftarrow 2^{\left\lceil \log_{2}N\right\rceil }$2: $H\left[n\right]\leftarrow FFT\left\{H\left[n\right],N_{fft}\right\}$3: $S_{lfm}\left[n\right]\leftarrow FFT\left\{S_{lfm}\left[n\right],N_{fft}\right\}$4: $S_{pulse}\left[n\right]\leftarrow IFFT\left\{S_{lfm}\left[n\right]\cdot H\left[n\right]\right\}$// Optimizing long vector convolution operations using the FFT operator.5: $S_{pulse}\left[n\right]\leftarrow \mathrm{abs}\left\{S_{pulse}\left[n\right]\right\}/\max\left\{S_{pulse}\left[n\right]\right\}$6: $D_{int}\leftarrow \;\mathrm{index}\;\mathrm{of}\;\max\left\{S_{pulse}\left[n\right]\right\}$// Obtaining delay value with sampling resolution.7: $S_{fit}\leftarrow S_{pulse}\left[D_{int}\right]-S_{pulse}\left[D_{int}+1\right]$8: $D_{frac}\leftarrow \left\{\frac{20\alpha^{2}-\alpha^{4}}{120}-S_{fit}\right\}/\left\{\frac{\alpha^{2}}{3}-\frac{\alpha^{4}}{30}\right\}$// Obtaining delay value with sub-sampling resolution.9: **Return** $D\leftarrow D_{int}+D_{frac}$

Due to the long duration of the LFM signal, direct convolution operations result in significant system overhead. Hence, we optimize the process by employing the Fast Fourier Transform (FFT) operator. Additionally, considering factors such as channel fading and impedance mismatch among circuit components, we incorporate amplitude normalization for the pulse compression results in the algorithm.

## 3. Error and Noise Performance Analysis

### 3.1. Error Analysis

The sub-sampling resolution of the proposed synchronization delay measurement algorithm is derived from the fitting of the pulse compression shape. Since obtaining a numerical solution for the fitting function in Equation (11) is challenging, a polynomial approximation is used instead, which introduces computational errors.

**Figure 3:**
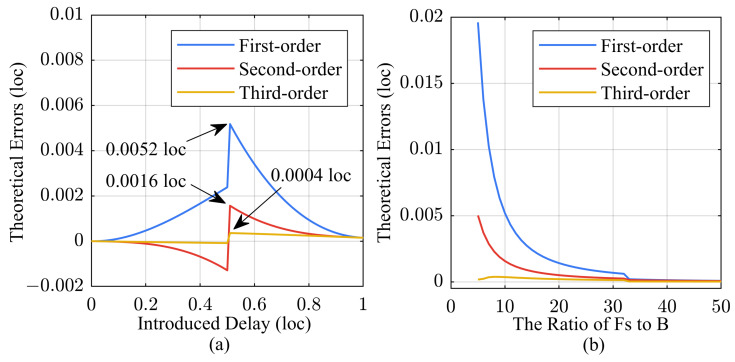
Illustration of algorithm errors for different fitting orders. (**a**) Sub-sampling resolution impact on algorithm error; (**b**) Signal bandwidth impact on algorithm error.

To validate the accuracy of the algorithm, we conducted simulation calculations on its sub-sampling resolution results. We introduced a delay value smaller than one sampling point into the signal, and the errors of the algorithm are shown in Figure 3.

Figure 3a displays the theoretical errors for three different fitting orders. The errors arise from the omission of higher-order terms in Equation (13), resulting in the largest deviation when the introduced delay is at 0.5 loc. The maximum error in the first-order fitting calculation is 0.0052 loc, and increasing the fitting order can significantly reduce the algorithmic error. Meanwhile, in Figure 3b, we evaluated the impact of the bandwidth of the LFM signal on the maximum error of sub-sampling resolution. In this article, we considered scenarios where the sampling frequency is 5–10 times the signal bandwidth, and achieved synchronous delay measurement with a resolution of 0.01 sampling points using basic first-order fitting. Certainly, users can adjust the bandwidth of the LFM signal or increase the fitting order to further enhance the resolution of the algorithm.

### 3.2. Noise Performance

In the ADC/DAC chains of multi-channel systems, it is challenging to eliminate factors such as component nonlinearity, thermal noise, and quantization noise from the converters. Particularly in systems with wireless transmission paths, additional components such as multipath fading and external electromagnetic interference are introduced, which significantly impact the quality of signal transmission in the chains. In this section, we evaluated the noise performance of the algorithm as follows.

**Figure 4:**
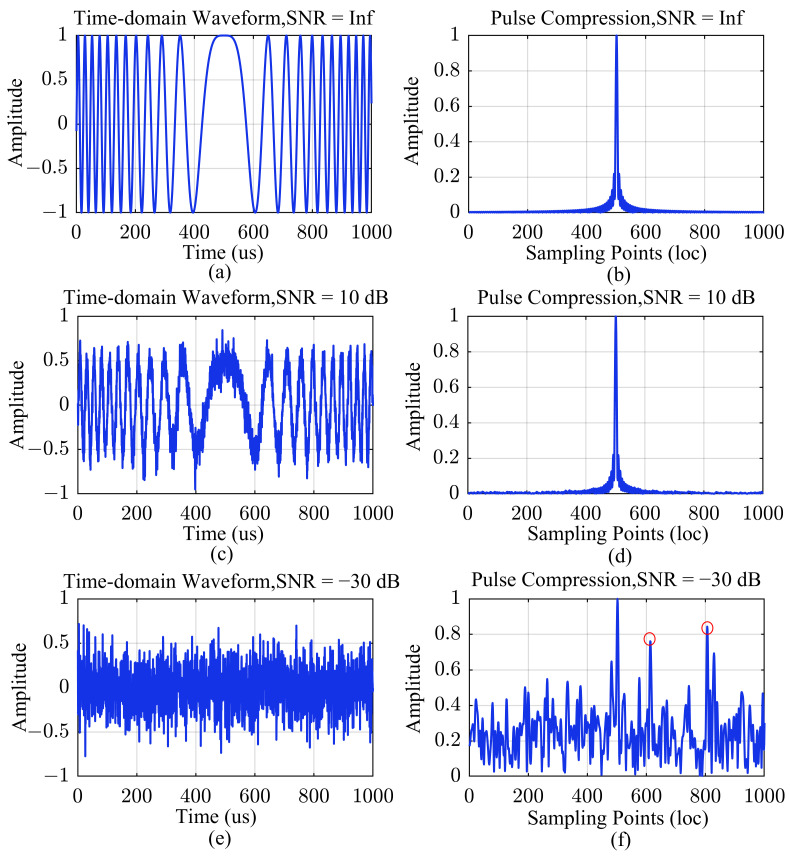
Illustrations of time-domain LFM signal waveforms and the corresponding pulse compression results for different SNRs. (**a**) LFM waveform with SNR = Inf; (**b**) Pulse compression result with SNR = Inf; (**c**) LFM waveform with SNR = 10 dB; (**d**) Pulse compression result with SNR = 10 dB; (**e**) LFM waveform with SNR = −30 dB; (**f**) Pulse compression result with SNR = −30 dB. The signal amplitudes in the figure have been normalized, and false pulse compression peaks are indicated by red circles.

Distinguished from the noises present in the ADC/DAC chains, LFM signals possess the characteristic of linear frequency variation over time. The pulse compression operation is mathematically equivalent to correlation computation. By accurately matching the LFM signal in the time domain, it enables effective signal extraction and suppression of noise impact. We can illustrate this issue through simulation to provide a visual understanding. The introduction of noise distorts the waveform but does not affect the peak of the pulse compression, as shown in Figure 4c,d. When the noise energy is much higher than that of the LFM signal, multiple false pulse compression peaks can cause the algorithm to misjudge the primary peak, as shown in Figure 4e,f.

**Figure 5:**
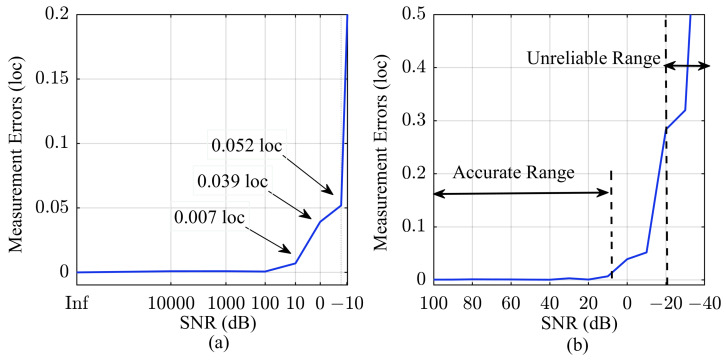
Illustration of the measurement errors for different SNRs. (**a**) Logarithmic display. (**b**) Detailed display of the SNRs ranging from 100 dB to −40 dB.

To demonstrate the effect of noise on algorithm accuracy, Figure 5 displays the measurement errors for different SNR levels. The results show that for the range where the SNR is greater than 10 dB, the error is less than 0.01 sampling points. We define this interval as the accurate measurement range, which is suitable for most wireless scenarios. Moreover, in scenarios where the noise power is close to or even overwhelms the signal, specifically when the SNR is between 10 dB and −10 dB, the algorithm exhibits an error of less than 0.1 sampling points. Furthermore, we define the interval with a peak misjudgment probability of greater than 10% as the unreliable measurement range.

## 4. Results

### 4.1. Experiment Platform

In this section, we validate the proposed chain synchronization measurement algorithm by applying it to a multi-channel radio system. The system is equipped with four independent transceiver chains that collectively perform tasks such as target detection, localization, and communication. The entire chain encompasses ADC, DAC, filtering, gain control, mixing, and other components. To ensure optimal synchronization among the chains, we measure the delay parameter differences of the ADC/DAC chains, and the results are used for subsequent compensation and calibration purposes.

**Figure 6:**
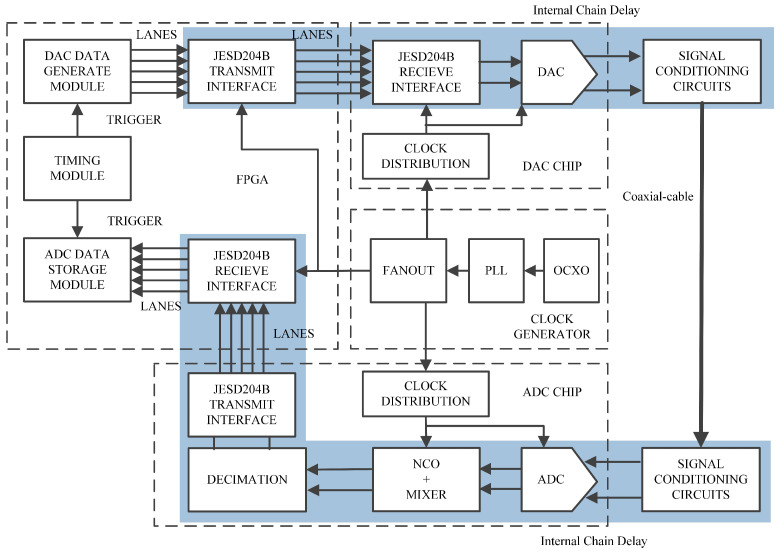
Single ADC/DAC chain measurement scheme, which can be easily extended to a scheme with multiple chains.

Figure 6 depicts the design scheme for measuring the chain delays of the system. The FPGA serves as the core logic device, encompassing functions like signal generation and data storage. Additionally, the data transmission between converters and FPGA is achieved through the JESD204B protocol. To mitigate the effects of clock jitter and offset, we have implemented an independent clock system based on an Oven Controlled Crystal Oscillator (OCXO), Phase Locked Loop (PLL), and Clock Fanout devices. This dedicated clock system ensures homogeneous clock inputs for both the sampling of converter devices and the operation of the logic device.

Figure 6 presents a comprehensive scheme outlining an ADC/DAC chain, which is also suitable for measurement scenarios involving multiple chains. The FPGA generates a specific LFM signal, which propagates through the DAC in the transmission chain. Meanwhile, the ADC samples and captures the electromagnetic waves in the receiving chain to acquire analytical data. This enables us to measure the signal’s propagation delay across the entire chain, effectively characterizing the synchronization differences between the chains. Based on this foundation, we can further estimate the internal chain delay by performing fitting operations, as depicted by the blue annotation in the figure. The parameters of the platform and the LFM signal used are shown in Table 1.

### 4.2. Chain Delay Measurement

In this experiment, we accessed coaxial cables of different lengths to change the signal propagation path and measure the delay differences among the four chains in the system. To minimize the impact of cable inconsistencies on the measurement results, we utilized a vector network analyzer to precisely measure the parameters of the three types of cables used, as shown in Table 2.

**Figure 7:**
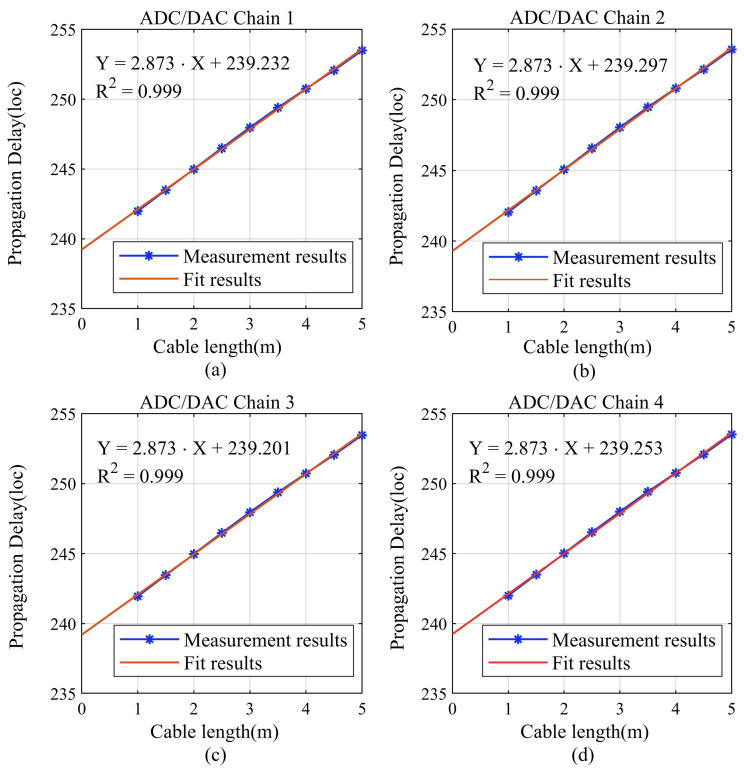
Measurement results of the delay differences among the four ADC/DAC chains. (**a**) ADC/DAC chain 1; (**b**) ADC/DAC chain 2; (**c**) ADC/DAC chain 3; (**d**) ADC/DAC chain 4.

Figure 7 presents the measured results of the delay for the four ADC/DAC chains, accompanied by a linear regression analysis of the data. The first-order coefficient of 2.873 indicated that the propagation delay increased by 4.788 ns for a 1.00 m increase in the length of the coaxial cable. We calculated the propagation speed of the LFM in the cable to be $2.088\times 10^{8}\;m/s$, which is consistent with the data in Table 2. The zero-order coefficient in the results signifies the internal delay of each ADC/DAC chain, indicating minor differences of less than one loc across the four chains. By compensating for the measured delay discrepancies, higher levels of synchronization performance can be achieved.

### 4.3. Precision Verification

**Figure 8:**
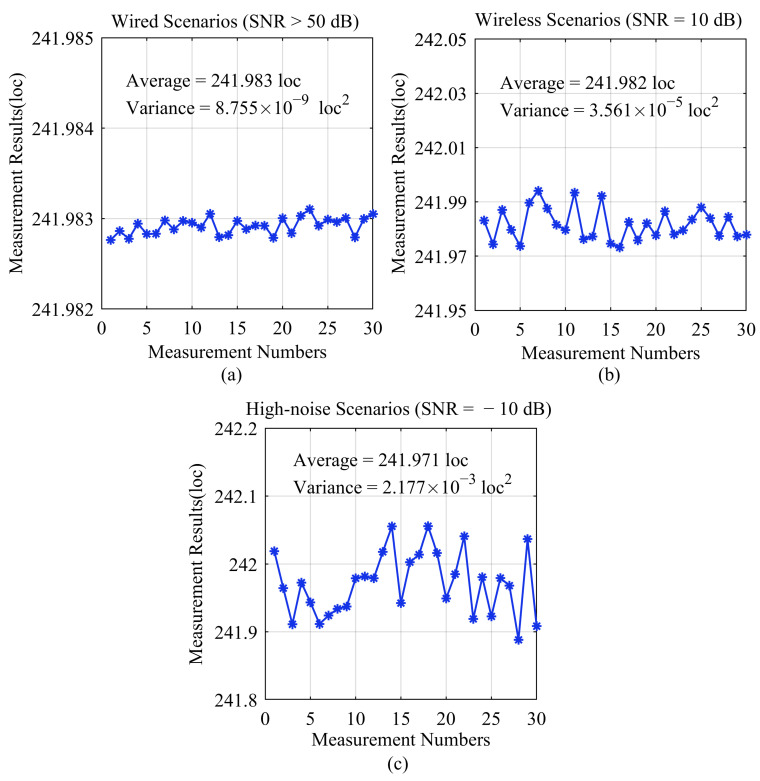
Statistical chart showing repeated measurement data for a specific chain delay. (**a**) Wired scenarios; (**b**) Wireless scenarios; (**c**) High-noise scenarios.

In the aforementioned process, we connected coaxial cables to the system to measure the internal delay parameters of the ADC/DAC chains. Additionally, the algorithm is also applicable to wireless scenarios. To validate the precision of the algorithm, we selected three scenarios with different SNRs, and the results are depicted in Figure 8. In wired scenarios with an SNR exceeding 50 dB, the algorithm exhibits a variance of $8.755\times 10^{-9}$ ${\mathrm{loc}}^{2}$ in its results, showcasing an exceptional level of measurement precision. Similarly, we conducted verification experiments in wireless and high-noise scenarios using the SNR values of 10 dB and −10 dB. The obtained variances were $3.561\times 10^{-5}$ ${\mathrm{loc}}^{2}$ and $2.177\times 10^{-3}$ ${\mathrm{loc}}^{2}$, which affirm the algorithm’s reliability in accurately measuring chain synchronization delays, even in challenging wireless and high-noise environments.

## 5. Conclusions

In this article, we address the synchronization issue in a multi-channel system. Existing research has mainly concentrated on the synchronization design for converter sampling or data transmission stages, making it challenging to accurately measure the delay value of the entire ADC/DAC chain. To this end, we propose a novel measurement algorithm that contributes by effectively mapping the shape of the pulse compression peak to sub-sampling resolution measurements. Experiments showed that the algorithm exhibits a favorable noise performance and is suitable for radio systems. In systems with an SNR greater than −10 dB, it can measure the delay values of all components in the ADC/DAC chains with sub-sampling resolution. The highest achievable resolution can reach 0.01 sampling points. Further, the results can be used to optimize the synchronization performance of multi-channel systems and for solving problems such as radar positioning deviation and sampling mismatch error. 

## Figures and Tables

**Table 1:** Parameters of platform.

Symbol	Detailed Explanation	Value
$f_{s1}$	DAC sampling frequency	1200 MHz
$f_{c}$	Carrier frequency	300 MHz
$f_{s2}$	ADC sampling frequency	1200 MHz
$f_{ddc}$	Digital down conversion frequency	300 MHz
dcm	ADC decimation factor	2
*B*	LFM signal bandwidth	100 MHz
*T*	LFM signal duration	10 μs

**Table 2:** Parameters of coaxial cables.

Connector	Cable Length (m)	Delay (ns)	Propagation Speed ($10^{8}\;m/s$)
SMA KJ	1.00	4.79	2.09
SMA KJ	1.50	7.18	2.09
SMA KJ	2.00	9.57	2.09

## Data Availability

Data are contained within the article.
